# Frail Older People Ageing in Place in Italy: Use of Health Services and Relationship with General Practitioner

**DOI:** 10.3390/ijerph19159063

**Published:** 2022-07-25

**Authors:** Maria Gabriella Melchiorre, Marco Socci, Sabrina Quattrini, Giovanni Lamura, Barbara D’Amen

**Affiliations:** Centre for Socio-Economic Research on Ageing, IRCCS INRCA-National Institute of Health and Science on Ageing, 60124 Ancona, Italy; g.melchiorre@inrca.it (M.G.M.); s.quattrini@inrca.it (S.Q.); g.lamura@inrca.it (G.L.); b.damen@inrca.it (B.D.)

**Keywords:** ageing in place, frail older people, living alone, self-rated health, health services access, general practitioner, barriers to use, Italy

## Abstract

Functional limitations, chronic diseases and frailty often occur in later life. These aspects become very challenging when older people age alone in place, thus needing support in the activities of daily living, and in this context, it is important they can access and use health services. The present study aimed to explore these issues in Italy. In 2019, 120 qualitative interviews were carried out within the “Inclusive Ageing in Place” (IN-AGE) project, involving frail older people living at home in three Italian regions (Lombardy, Marche, and Calabria). A content analysis and some quantifications of main statements are presented. Results showed that the majority of seniors report poor self-rated health (SRH), suffer from many chronic diseases, and mainly use the General Practitioner (GP) and Medical Specialists (MSs), even though long waiting list in the public sector and high costs in the private one act as barriers to access health services. Complaints regarding GPs mainly refer to the almost exclusive provision of prescriptions and the lack of home visits. Some regional peculiarities highlighted a better overall context in the north than in the south, especially with regards to the public health sector. These results can provide useful insights for policy makers, in order to deliver health services assuring frail, older people the continuity of assistance needed at home.

## 1. Introduction

In Italy, the number of older people aged 65 years and over is about 24% of the total population, as of 1st January 2022 [1], and is expected to rise to 30% by 2040 [2]. Across the European Union (EU) (which includes 27 countries), this percentage is 21%, with Italy (having the highest value across the EU) followed by Finland, Greece and Portugal (22–23%), while Luxembourg and Ireland having the lowest shares (15%). Also, median age is highest in Italy compared to the EU member states average, which is 48 and 44 years, respectively [3]. Moreover, in this country, about half of all people living alone are aged over 65 years. In particular, in the period from 2017 to 2021, the number of couples with children has decreased (from 33% to 31%), whereas the number of people aged 65 and over, and living alone, has increased overall (from 47% to 50%), more so for men (+4%) than women (+2%), even though the latter live without family members more frequently than the former (32% vs. 63% in 2021, respectively,). Moreover, older people living alone are more concentrated in the south of Italy (52%), and in small/rural municipalities with up to 2000 residents (55%) [4].

Overall, seniors reported severe functional limitations [5]. Indeed, physical and cognitive limitations often occur in later life and are a predictor of the overall degree of older adults’ frailty [6]. Frailty is a common condition in old age, being a multidimensional syndrome that implies extreme vulnerability to adverse medical consequences, affecting several domains: e.g., physical, cognitive, clinical, psychological and social. In particular, the following aspects are crucial: health/functional status (e.g., chronic diseases, limited functional abilities); mobility (e.g., loss of autonomy in mobility); cognition (e.g., memory/cognitive impairment); nutrition (e.g., malnutrition); physical activity (e.g., sarcopenia); and the socio-economic context (e.g., poor social network, low income) [7,8,9]. Functional frailty in particular is generally linked to difficulties in performing the basic and instrumental activities of daily living (ADLs and IADLs) [10,11]. Ageing is also correlated with increased risk of illness and multimorbidity (as the co-existence of two or more chronic diseases in one person); the consequent higher risk of hospitalization [11,12,13]; functional disability and need of daily assistance [14]. In multimorbid older adults, the consumption of several prescribed drugs (polypharmacy) is higher, with sometimes up to five or more drugs taken daily [15]. When comorbidities increase, the level of independence/ability in performing the activities of daily living, both simple and complex, decreases [11]. Multimorbidity, disability and frailty [16,17], as well as living alone, is associated with both poor mental and physical health, loneliness and mortality [18]. The ageing population is also often linked to an increased use of healthcare [19,20], due to the relationship between ageing and the worsening of pathologies [21]. Moreover, since frailty represents a risk factor for illness in older people, this in turn leads to crucial challenges in healthcare policies. Indeed, some authors have shown that frail older adults are more likely to use community services [19,22], and that frailty implies high healthcare expenditures [19,23,24]. It is estimated that around 70–80% of global health resources are currently spent on the management of chronic disease, which represent 80% of all diseases in the world. Illnesses such as heart failure, respiratory failure, diabetes and hypertension affect 80% of people over the age of 65 worldwide, which have important negative consequences on their autonomy in daily activities [25].

Regarding the health status of seniors in Italy, and considering the 2019 data referred to [26] (year of the survey in our study), 32% of those aged 65 and over, and 48% of those aged over 85, have serious chronic diseases and multimorbidity. The most common pathologies are osteoarthritis (48%) and hypertension (47%), followed by heart disease (19%) and diabetes (17%). The share of those with at least one severe chronic disease is 43% with regards to the over 65 years old, and 48% for those in the age range of 75–84 years. With increasing age and multimorbidity, the share of older Italians reporting a positive opinion on their health status decreases, from 42% for those aged 65–74 years, to 26% for people aged over 75 years [27]. 

Regarding the use of both public and private health services, according to ISTAT [26], and in the year preceding their interview, almost all Italian seniors aged 65 years and over (90%) contacted their General Practitioner (GP) (family doctor) at least once. Moreover, 66% had visits from Medical Specialists (MSs); 48% underwent specialist examinations; and about 14% turned to a rehabilitation professional at least once. These figures rise. Respectively, to 68%, 49%, and 15% at the age of 75 and over. In addition, older people aged 65 years and over account for 46% of inpatient admissions and 60% of pharmaceutical expenditure [2]. However, barriers hampering the access to health services are reported [26]. In 2019, 10% of older people gave up at least one health service (e.g., medical visits, clinical analysis, diagnostic tests) for economic reasons, and about 20% had to postpone healthcare services due to long waiting lists. Difficulties in accessing health services are mainly reported by older people aged 75 years and over. Previous ISTAT data [28,29] highlighted these barriers, with consequent waiver or delay in the use of services mainly due to long waiting lists (22%), no available means of transport (8%), and economic reasons (8%). 

In Italy, territorial/regional differences in access to health services should be considered too. In the south, compared to the north, the share of seniors aged 65 years and over who turned to MSs is lower (63% vs. 67%) [26], whereas difficulties in accessing health services (and related renunciation) are higher. The territorial gap lists long public waiting lists (16% in the north, 21% in the center, and 24% in the south) and economic reasons, in particular for private medical examinations and treatments (3% in the north, 7% in the center, and 9% in the south) [26,30]. In particular, previous ISTAT data [28,29] evidenced barriers such as long waiting lists, no available means of transport and economic reasons, as being more crucial in sparsely populated areas in the center (28%, 12% and 6%, respectively), and in the south (25%, 12%, 11%), than in the north of Italy (18%, 4% and 4%).

The “health picture” of Italian seniors, as depicted above, clearly indicates that when frail older people are ageing alone at home, and have multimorbidity and functional limitations compromising the activities of daily living, the possibility of accessing and using health services is crucial. Beginning from these considerations, this paper aimed to answer the following research questions: (1) How do frail, older Italian people with physical limitations, and ageing alone at home, perceive and report their health status? (2) Which health services, both public and private, do they use, including their GP? (3) How do frail older people with physical limitations perceive their relationship with their GP? (4) Which barriers hamper their use of health services? (5) Are there regional differences in this regard? Taking these questions into consideration, it is hypothesized that a great number of older people with functional limitations could perceive as having a poor health status, use several health services and experiencing obstacles in accessing them; this was especially true in southern Italy, where the efficiency of such services is often low. Regarding their relationship with GPs, it was assumed that the care and availability provided by home visits could positively impact seniors’ perception of the service. The analysis of these aspects can provide insights for both policy makers and the National Health Service (NHS), in order to better organize and deliver health services for frail older people, continuing to consider their complaints or appreciation, and thus further improving what seems to be working well, and adequately changing what is not.

## 2. Materials and Methods

### 2.1. Study Design and Participants

Results come from the “Inclusive Ageing in Place” (IN-AGE) qualitative study, which was carried out in 2019 in three Italian regions: Lombardy (north), Marche (center) and Calabria (south/mid). In the following order, these regions are representative of high, medium and low levels of socio-economic development of the country [31,32]. In each region, one medium-sized urban city (100,000–200,000 residents) [1], and one inner/rural area [33], were included. The urban cities were: Brescia, Ancona, and Reggio Calabria; and the rural areas were Oltrepò Pavese, Appennino Basso Pesarese e Anconetano, and Area Grecanica. Overall, the most fragile locations were detected where there was a greater presence of older people living alone and in public housing (*Edilizia Residenziale Pubblica*-ERP); where there was high unemployment; low educational levels; and where there was a low provision of public services [34]. A purposive sample [35] was built, with 72 respondents from urban cities (24 each), and 48 from rural sites (16 each), with a total of 120 older people (40 in each region). Inclusion criteria for participants were as follows: older people aged 65 years and over (both men and women) living alone at home, or with the support of a Personal Care Assistant (PCA); mobility within the home and outside, with help (from a person or aids); no cognitive impairment, in order to answer interview questions independently; and the absence of very close family members (living in the same urban block/rural building) giving support for daily activities. Our study analyzed frailty as a condition linked to ageing, limited functional abilities, reduction of independence and the increased need for support [36]. Recruitment was carried out with the support of operators from local sections of voluntary associations (e.g., Auser, Anteas, Caritas), and from home care service (SAD, *Servizio di Assistenza Domiciliare*), who contacted and informed potentially eligible participants on the purpose of the study, and collected preliminary information in order to communicate the contact details of seniors (address and telephone numbers) to interviewers. A cognitive test was not used as preliminary screening, but the assessment of the participants’ status was assured by recruitment channel operators (e.g., operators of SAD), by means of their own/verified information in this regard, then further confirmed by the interviewers after speaking to the families/caregivers of the older people being interviewed.

### 2.2. Data Collection, Measures, and Ethical Issues

In May-December 2019, six researchers (two for each region, mainly psychologists and sociologists) carried out qualitative face-to-face interviews using a semi-structured interview/topic guide, with questions regarding: socio-demographic aspects; health status/diseases and functional limitations; care arrangements and use of health and social services; and the housing and economic situation of respondents. The topic questions were adapted from previous similar studies [37], with the addition of validated scales to detect functional limitations, i.e., ADLs and IADLs scales [38], and questions on both sensory and mobility limitations [39,40]. The Ethics Committee of Polytechnic of Milan (POLIMI, Research Service, Educational Innovation Support Services Area, authorization n. 5/2019, 14 March 2019), gave approval, and a written informed consent was signed by each participant before being interviewed. Participants were fully reassured on anonymity and privacy of the information collected, according to ethical issues stated by *The EU General Data Protection Regulation (GDPR) n. 679*, of 27 April 2016 [41]. 

### 2.3. Data Analysis

The interviewers audio-recorded narratives and transcribed them verbatim, by replacing the identity of respondents (i.e., name, address, telephone number) with alphanumeric codes [42]. The steps of the Framework Analysis Technique [43,44] were applied, by performing the following activities: reading the transcribed narratives; identification of macro-sub-categories/themes; indexing-labelling; construction of a thematic chart; interpretation of contents. A thematic content analysis was carried out [45]. This task was done manually, without the support of software, as also allowed by the literature [46,47]. It was, however, facilitated by using interview questions as a preliminary conceptual framework/guide, since they were based on theoretical categories relevant to the study, and were drawn from the overall literature and experience of researchers [42,48]. Moreover, each category/theme was assigned a cell color, and this color-coded process allowed for grouping data [49]. The final codes were thus categorized according to respective similarities and differences. All researchers of the consortium discussed the appropriateness of reading/coding the contents [42]. For the analysis, the following main categories/labels were examined: (Table 1). 

With regards to the overall “health status”, the question asked was: “How do you rate your health in general? Do you have any disease?” The respondent’s perception, i.e., “self-rated health” (SRH), was categorized as “poor/unstable” or “good/acceptable” when explicitly referred to by the interviewees, or when deduced from more generic statements, such as: “I feel bad/I have many physical complaints,” or “I feel well overall.” Statements relating to the objective state of health were expressed through one or more specific pathologies. Similarly, their relationship with the GP was classified as “good” or “bad” when explicitly referred to by the interviewees, or deduced from complaints or appreciation reported in this regard, and classified ex post, as described in the related column “label”. Qualitative dimensions are presented in figures elaborated with Microsoft Excel 2019 (Microsoft Corporation, Washington, DC, USA), by quantifying statements as absolute values, with some regional differences when relevant. Original verbatim quotations are also included [50], and coded with the first three initials and progressive interview number (1–40) of the respective region (LOM = Lombardy; MAR = Marche; CAL = Calabria). Not relevant omissions were put within round brackets, and some words/terms were addedwithin square brackets, in order to help the understanding of quotations. Further details on the Methods (setting, sampling, participants, data collection, measures and data analysis) can be found in a previous publication [42], from which the section “Materials and Methods” has been partly adapted. 

## 3. Results 

### 3.1. Sample Characteristics

Table 2 presents the main socio-demographic characteristics of the sample. 

Respondents consist of 120 older people aged 85 years and over; living alone without family members or a PCA; women; those with low educational levels; widowed; and people with a mild/moderate level of physical limitation. More information on the sample is available in Melchiorre and colleagues [42]. 

### 3.2. Health Status of Older People

#### 3.2.1. How Older People Perceive and Report Their Health

**Figure 1:**
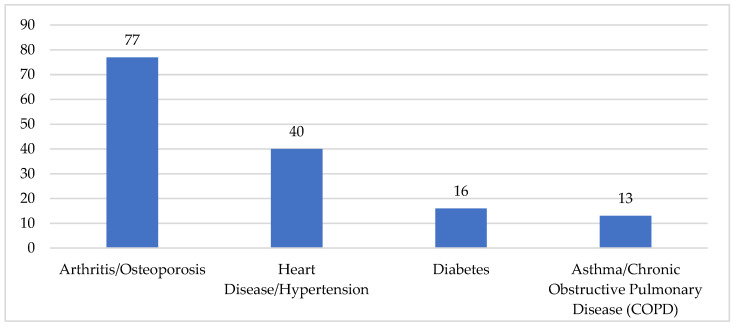
Main diseases (*n*). Some respondents reported more than one category of main disease.

The interviews reveal 78 older people with poor-unstable health (65%), and 42 (35%) who, despite some “problems” (sometimes several), refer to themselves as having good-acceptable health. In essence, the latter, despite some “physical ailments” still consider their health to be fairly good. Various chronic diseases are also reported, also in combination (i.e., multimorbidity) (Figure 1).

In the main, cases of arthritis/osteoporosis (77) were reported, with musculoskeletal problems and difficulty in walking, pains in the legs and feet (sometimes following falls/fractures), in addition to unsteady balance, weakness/unsteady gait, and arthroplasty. Also, heart disease/hypertension (40 cases), diabetes (16 cases), and asthma/chronic obstructive pulmonary disease (COPD) (13) were referred to. No particular regional differences emerged in this regard.

#### 3.2.2. Poor-Unstable Health

The interviewees’ original narratives help to better define the results summarized above. Those who report poor health often describe it as a result of multiple pathologies, which greatly compromised their autonomy.

*My health is bad! (…). I have arthrosis, four herniated discs, circulation problems and depression. (CAL_32)*

*I rate my health very badly. I have a lot of problems. I have no breasts, I have diabetes, I take insulin four times a day. (CAL_17)*

The progressive loss of autonomy represents a crucial problem, and difficulties in walking, especially outside the home, emerged. It should be considered that walking problems are often amplified if a paving with cobblestones prevents older people from being able to walk well.

*I can hardly walk! I cannot go for walks with my friends! I also struggle to get out of bed. (LOM_36)*

*I walk badly, the external flooring is a problem, it is also a question of safety. (MAR_39)*

Some seniors also feel very old, which amplifies all complaints, especially due to chronic pathologies.

*My health is worsening! The doctor told me that my diseases cannot be solved, since they are chronic. (CAL_32)*

*I have heart and moving problems (…). As age increases, health problems also increase. (LOM_5)*

Sometimes health problems started very early, at a relatively young age, leading over time to a severe disability in old age.

*My [health] problems started when I was 40 years old. Currently I am a disabled person. People tell me I am strong, but it is not true, I force myself for appearing so! (CAL_2)*

Other times the health is compromised by a period (three-four years) of assistance from a family member, e.g., a spouse.

*My health is bad, I am really in bad shape. I spent last three years caring for my ill husband [then died]. In that period I really led a crazy life, I slept an average of four hours a night and it was really nerve-wracking. (LOM_29)*

#### 3.2.3. Good-Acceptable Health 

Other older people, despite some pathologies, are “satisfied” with their health, in light of their age, and report only some “ailments”.

*I am a woman of almost 80 years, thus I rate my health accordingly. I have some physical complaints but nothing so serious. The knee is the one that can give me most problems. I have severe osteoarthritis but I feel overall stable. (MAR_36)*

*I consider my health to be good, even if I have pains in my back, hands, legs, and I cannot carry weights. (MAR_18)*

Some seniors also appreciate their own (more or less) acceptable health, since they know older people with a worse health status, and thus they feel “lucky” to some extent.

*I try to manage my health as much I can. At my age there are people who are in wheelchairs, or enticed. There are several in my city. (CAL_29)*

However, the important thing is to still have a “working head/brain” and to be mentally lucid, despite various physical problems.

*I am happy because I am still mentally lucid. And this is not a small thing, it is a great help. If someone begins stammering, and he is not able to speak anymore, it seems he is no longer of any “use”. (CAL_1)*

### 3.3. Public and Private Health Services Used by Older People

It seems useful to premise that some interviewees with particular health problems, could actually use even more services than those reported. For example, a lady with sight and hearing problems declares she does not know exactly which or how many health services she benefits from, besides the GP, since this is provided by her daughter.

*I cannot say which services I use [besides the GP], because it is my daughter who does everything (…). I see and hear nothing. I am not able to contact services and so on. (LOM_3)*

It should also be emphasized that, from this narrative, it emerged that what unfortunately often happens among older people who live alone and are widowed, there is no longer any desire to be treated. One interviewee, after the death of his wife (two years earlier), had stopped taking care of his health, abandoning and reducing checks and controls in this respect.

*When my wife was still alive, I did blood tests every six months. Since she died I stopped. I do not see the physiotherapist anymore. I go sometimes to the cardiologist and urologist because my GP insists. (CAL_16)*

#### 3.3.1. Public Health Services

**Figure 2:**
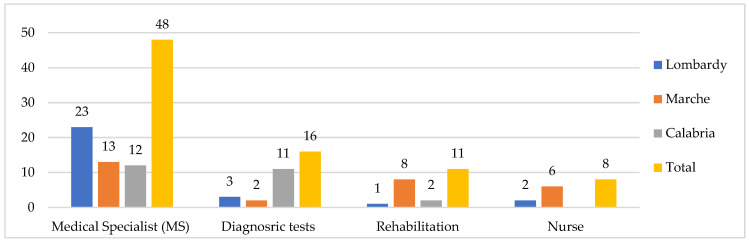
Public health services used by older people, by regions (*n*, GP excluded). Some respondents reported more than one type of listed public health services. MS was mainly cardiologist, physiatrist, orthopedist and diabetologist. Diagnostic tests were mainly blood/urine tests, ultrasound, electrocardiogram (ECG), and computed axial tomography (TAC). Also, nurses at home for injections, withdrawals and catheter management.

All the interviewees needed the GP (not shown in the figure below), but the MS is also fairly frequently requested (48 cases, especially in the north). Diagnostic tests, mainly in hospital (16 cases, especially in the south), rehabilitation and nursing care (11 and eight cases, especially in the Marche region) follow (Figure 2).

Regarding the use of the public health sector, the interviewees do not report particular details, but emphasize the mediation of their GP. In Italy, medical visits and examinations, when required by MS, must be authorized by the GP if patients want to use the public health sector.

*I go to the public cardiologist with my GP’s prescription. (CAL_25)*

*My GP sent me to a public cardiologist, because I have high blood pressure, and also she sent me to the public physiatrist. (LOM_15)*

#### 3.3.2. Private Health Services

**Figure 3:**
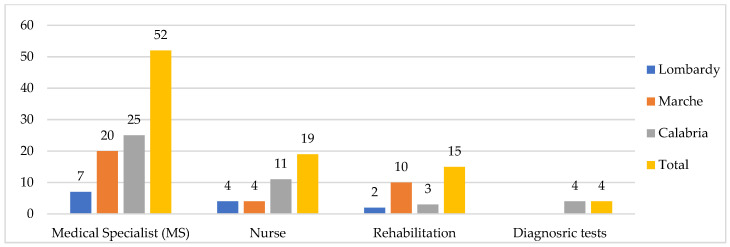
Private health services used by older people, by regions (*n*). Some respondents reported more than one type of listed private health services. MS was mainly cardiologist, physiatrist, orthopedist and diabetologist. Also, nurses at home for injections and withdrawals. Diagnostic tests were mainly blood/urine tests, ultrasound and electrocardiogram (ECG).

Overall, interviewees turn slightly more to private, than public services. The most requested private health service is the MS (52 cases, especially in Lombardy region), followed by nurses and rehabilitation therapists (19 and 15 cases), and four cases of diagnostic tests, which are, however, reported only in the Calabria region (Figure 3).

At the regional level, there is a lower use of private MS in Lombardy, when compared to the other two regions, but there is also a greater recourse to the rehabilitation therapist in Marche, and to nursing care and home blood tests in Calabria.

*When I need [blood] analysis, a nurse comes home from the laboratory, and I have to pay for this private service. (CAL_3)*

However, it should be noted that, in the Calabria region, sometimes it is not necessary to request the assistance of a nurse, in particular for the injections, if a family member is able to give them “informally”.

*My niece gives me the injections and I do not need a nurse. Even if I get a fever and I have to take penicillin, my niece is always available. (CAL_38)*

### 3.4. Barriers for Using Public and Private Health Services

It should be noted that “barriers” for using the GP will be analyzed in more depth in a subsequent paragraph. Furthermore, problems in accessing public and private health services were reported both by interviewees who use them with difficulty, and by others who do not use them because of certain “impediments”.

#### 3.4.1. Public Health Services

**Figure 4:**
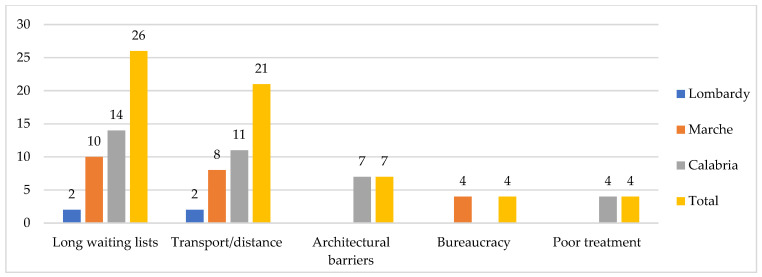
Barriers for using public health services, by region (*n*). Some respondents reported more than one type of listed barriers.

The greatest problem in using public health services stem from long waiting lists and the geographical distance of the health facilities from one’s home, with the subsequent difficulty in reaching them, and the consequent need for accompaniment/transport (26 and 21 cases). Overall, seniors in the south mostly reported these problems, in addition to various cases of “poor treatment” (e.g., several hours of waiting for treatment), and architectural barriers preventing easy entry into health facilities. In the center of the country, the excessive bureaucracy related to various administrative practices (in order to benefit from the necessary services) is also highlighted, whereas in the north, only two cases for both long waiting lists and distance emerged (Figure 4).

#### 3.4.2. Private Health Services

**Figure 5:**
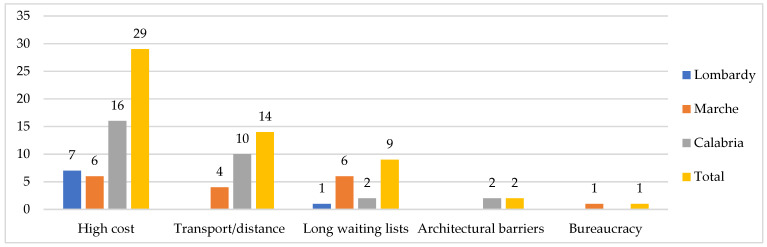
Barriers for using private health services, by region (*n*). Some respondents reported more than one type of listed barriers.

With regard to private health services, major barriers were their cost (29 cases), transport need (14 cases), and long waiting lists (nine cases). Few barriers in Lombardy were reported, and were almost exclusively related to the cost (seven cases). Conversely, major problems for cost and transport in Calabria emerged (16 and 10 cases), and the presence of long waiting lists for seniors in the Marche region is reported (six cases) (Figure 5).

#### 3.4.3. Specific and Common Barriers

*High Cost*

The cost issue emerged exclusively for private services, in particular for MS visits. In some cases, it is even necessary to give them up for economic reasons.

*With my low pension I will not be able to treat myself properly. As long as my husband was alive and there was also his pension, we were able to purchase private health services, MSs visits. But currently I cannot anymore! (MAR_25)*

When older people do not give up private services, they need to give up something else, or resort to savings, or else it is necessary to get financial help from family members.

*I have not given up on MS visits but I need to be suitable for eating, and even a little warmed rice can be enough. (CAL_20)*

*I spent a lot of money [for treating health]. All my savings were necessary in this regard (…). MSs want lots of money (…). (CAL_19)*

*My son also helps me [financially] to go to the MS. (CAL_33)*

*Long Waiting Lists*

Long waiting lists represent a crucial problem of public health service. Sometimes it is necessary to pay for private services, in order not to wait too long for public healthcare.

*I had to book a visit with the public urologist in January to have it in June! I had to wait for six months! (CAL_16)*

*To get health services, when you pay you can have them quite immediately. If you do not pay, you have to wait at least one year. (LOM_12)*

The situation becomes critical if seniors cannot wait so long for a public service, but neither can they pay for a private one.

*I had to wait four months to do physiotherapy, but I cannot wait so long. I need a course of massages but to have them I must wait again! (…). If I want them immediately I have to pay, but how can I pay for it? I cannot pay! (CAL_15)*

It can also happen that the service older people need is provided too late, and in the meantime, their health can worsen.

*It is so long that I have booked a visit, and I am still waiting to do it. In the meantime, my health has deteriorated. (MAR_9)*

However, the problem of waiting lists seems to emerge, in some (few) cases, even when seniors try to book a private medical service.

*I had to go to the ophthalmologist, and in order to have a public free visit I should have waited for nine months. Thus I decided to go to a private doctor. I paid 150 EUR but however I waited for about four months. In any case, for medical visits we have to wait for months and months! (MAR_25)*

*Architectural Barriers*

These barriers are reported more in the public than in the private sector (respectively, seven and two cases, only in the Calabria region). Indeed, the former sector seems less equipped/adequate in this regard, and sometimes laboratories are “on the upper floors”, with malfunctioning elevators.

*There are many stairs, I use a wheelchair, where can I go? (CAL_15)*

*Often the lift does not work and you have to go up to the fourth floor where there is the analysis laboratory. You should see how many people with crutches and sticks drag themselves up to the fourth floor, whereas at the first floor there are the administrative offices! (CAL_2)*

*Bureaucracy*

Even excessive bureaucracy (reported in five cases in the Marche region, of which four were in the public sector) can lead to a long wait to access, in particular, public health services. For instance, several months are necessary to obtain documents allowing the testing of a walker with trolley.

*Life is not easy for older people, since they need to manage a lot of bureaucracy when they need a health service! Doctors send you left and right, here and there, everywhere, and always some document seems missing! (MAR_14)*

*My nice has suffered a lot to have documents for testing my walker with trolley! (…). Once she had them from the GP, she had to make a lot of steps among outpatient clinics to have the necessary authorization! (MAR_5)*

*Poor Treatment*

Sometimes, poor treatment of public health facilities is also reported (four cases in Calabria), due to various disservices, e.g., several hours of waiting for treatment or checks in emergency room, and little attention from medical and nursing staff.

*Last summer I had a serious nosebleed! I went to the emergency room and I waited a long time, five hours on a wheelchair despite my old age. It was such a bad experience! (CAL_24)*

*Some doctors have rude attitudes! Some health services are pitiful! (CAL_16)*

*Distance and Transport*

The problem of long distances from seniors’ homes to access services, and the consequent need for accompaniment and transport, seems quite independent of either the public or private connotation of the service itself. For a private visit, sometimes it could be necessary to reach a city outside the region where older people live, and the cost of transport must be added to the medical one.

*Services are generally too far away, and I cannot go alone! (CAL_32)*

*When I need a visit, I go distant from my home, and thus I pay generally 70 EUR [for the trip], in addition to the cost of the visit. (MAR_34)*

The question arises in particular if older people have difficulty in walking, e.g., they use a cane and it is complicated to take a bus.

*The main problem is that I cannot move easily, and doctors in general are distant. (CAL_14)*

*The bus is not suitable for older people. It runs too fast, stops are few and inadequate, timetables are lacking. If you need to take a bus, you go out crazy, since you do not know where you have to go! (CAL_7)*

In the absence of alternatives, seniors are often forced to ask to be accompanied by a family member (especially children). When family members cannot accompany their older relatives, someone else is asked for help, for a small fee. Seniors also resort to volunteering for a “symbolic” fee. However, as for the cost, the problem of transport can induce older people to give up some health services, especially if they do not have available help for moving.

*The problem is the distance and therefore I have to go with the car. Usually I go with my son. When he cannot help me, I call someone [acquaintance] and ask him for a ride in order to have for instance blood analysis in the hospital (…). I pay at least EUR 20 for this. (CAL_38)*

*When I need to make blood analysis, I pay five EUR to the Auser [voluntary association] for transport [to the hospital]. (LOM_34)*

*I rarely go to the MS since I need someone who can accompany me! (CAL_33)*

### 3.5. The Relationship with GP: Role, Presence-Absence

#### 3.5.1. Prescriptions, Phone Calls and (Often) Little More

All the interviewees use their GP, mainly for the prescribing of drugs, MS’s visits and diagnostic tests. In-person contacts are not frequent, and are often supplemented/replaced (when possible) by phone calls, or activated through family members.

*I go to my doctor only for prescriptions, and for the referrals, every 2 months. (LOM_28)*

*I call my doctor by phone to communicate some health problem and have prescription. (CAL_9)*

*My children go to the GP for prescriptions, I do not go. (CAL_20)*

#### 3.5.2. Happy and Un-Happy GP’s Patients

**Figure 6:**
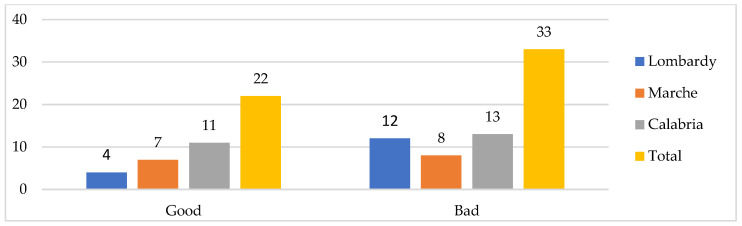
Relationship with GP (*n*).

Not all the respondents made explicit/spontaneous references to the relationship they have with their GP (there was no specific question about it). However, in several parts of the narratives, various opinions emerged on the role and presence-absence of this health professional, and also several problems with regards to accessing the related assistance. Among those who reported an opinion (55 cases, 16 in Lombardy region, 15 in the Marche and 24 in Calabria), a bad relationship mainly emerged (33 cases, vs. 22 good cases), especially in Lombardy and Calabria. However, in the latter region, cases of good relationships also prevail (half of the total), compared to the other contexts. (Figure 6).

#### 3.5.3. When the Relationship with GP Is Bad 

**Figure 7:**
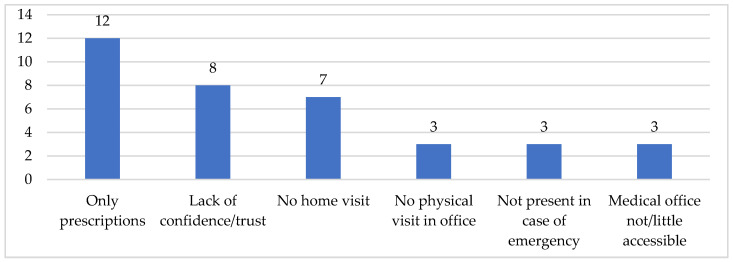
Reasons supporting a bad relationship with GP (*n*). Some respondents reported more than one type of listed reasons.

When the relationship is defined as unsatisfactory, the low availability-visibility of the GP mainly emerges, and it is declined above all as follows: only prescriptions of both drugs and medical examinations (12 cases); lack of confidence/trust (eight cases); and no home visits provided (seven cases). There are then fewer cases of low propensity to “actually/physically” visit the GP by older people who report symptoms; “invitation” to contact the emergency room/medical service in case of urgent need; and medical office not/rarely accessible (Figure 7).

An in-depth analysis of the results, through the statements referred to in this regard, also highlights certain regional prevalence/specificity.

*GP “Writes” Only Prescriptions*

The main prescriptive function is a complaint reason for some (mainly in Calabria, eight cases). In particular, a senior defines his GP a “pen doctor”.

*The GP just writes something! In short, only prescriptions! (CAL_19)*

*The GP is a pen doctor, because unfortunately the real efficient family doctor is no longer available. (CAL_11)*

*Lack of Confidence/Trust with GP*

Sometimes, inaccurate diagnoses, in addition to a poor caring attitude, generated distrust in the GP (mainly in Calabria, four cases), and in turn the request to be assigned to another GP. Even the pharmacy is preferred in some circumstances, for example for measuring blood pressure.

*I use GP in few cases, for medicines, for prescriptions, because sometimes he was not punctual in his diagnosis and therefore I do not trust on him so much. (CAL_2)*

*My son went to the doctor for a referral. The doctor did not ask him about my health status. Even though I have had this GP for a long time, just today I have changed the family doctor. (CAL_35)*

*I go very little to the GP. I go to the pharmacy more frequently, also to take my blood pressure for free, and this gives me more satisfaction. (MAR_29)*

*GP does not Visit at Home*

Respondents strongly disagree with the lack in provision for home visits (mainly in Lombardy, five cases). Moreover, some GPs do not even know the address of their patients. An exceptional event, therefore, seems necessary in order to have the doctor visiting at home.

*Home visits? They do not exist! (LOM_36)*

*My GP does not yet know where I live! (LOM_15)*

*If I call my family doctor and ask for a home visit, I should tell him I am dying, otherwise he does not come. (MAR_22)*

*GP does not Provide Physical Visit in Office*

In three cases, seniors go to the GP’s medical study, but are not actually/physically examined. Moreover, when there are specific health problems, the GP usually prescribes only some diagnostic tests and/or some other specialist visit.

*When I feel bad I go to the doctor, but he does not visit me (…). I had chest pain and he sent me to do the electrocardiogram. My eyes were watering, and he sent me to the ophthalmologist. I must ask him to take my blood pressure, otherwise he does not provide this. When I bring him results of some medical test, he only writes them on the computer, and nothing more! (MAR_16)*

*For Emergencies “Turn Elsewhere”*

When GPs sometimes suggest the patient should directly contact the emergency room/medical service, in case of urgent need, respondents seem very upset (only three cases in the Lombardy region).

*Despite all serious health problems, I had last year, when I called my GP he responded I had to go to the emergency room. I went there several times last year. (LOM_16)*

*My GP tells me to call the emergency medical doctor if I need, since I have too much health problems and he cannot manage them all! (LOM_1)*

*Medical Office Not/Little Accessible*

Further emerging complaints (only three cases in Calabria) do not refer specifically to questionable behavior of the GP, but rather to the difficulty of reaching his/her medical practice, due to geographical distance, especially for older people living in rural areas, or due to the lack of access for disabled individuals. These circumstances also negatively affect the relationship with this healthcare professional.

*I live in a rural area and the GP is available only twice a week. Thus, if I need him in other circumstances, I must be accompanied by some family member to reach his second medical study in another municipality. (CAL_37)*

*I move with a wheelchair. When I go to the [doctor] office I wait [at the entrance to the building] on the street for the secretary who opens the office and tell her what drugs I need. The building unfortunately does not have a proper access for the disabled! (CAL_11)*

*Regret for a Previous GP*

Narratives also reveal (in a couple of cases) regret of some seniors for missing a “historical” doctor, for losing a consolidated and trusted “family” relationship. The new doctor is not always like the previous one.

*In five years I have changed three GPs. Now a new one has come but he does not know anything about my health status. Each doctor has his own methods, and with all these changes patients get worse. (CAL_32)*

*My GP passed away at the end of February, then I had to take another one and yesterday I saw him for the second time (…). I still have to get over this sudden gap. It is not easy! (LOM_13)*

#### 3.5.4. When the Relationship with GP Is Good 

**Figure 8:**
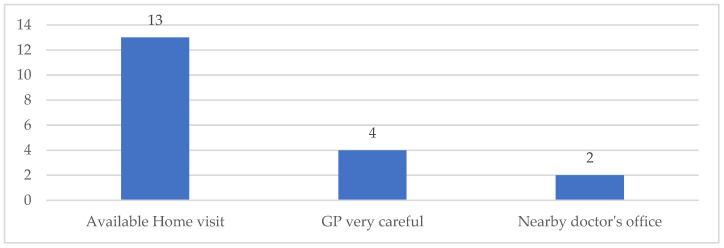
Reasons supporting a good relationship with GP (*n*). No reason was reported in three cases.

The possibility of receiving a home visit is much appreciated by the respondents and positively affects their opinion on the relationship they have with the GP (13 cases). In addition, the presence of a very caring GP, and the doctor’s office being geographical nearby is appreciated (Figure 8).

As in the case of bad relationships, the examination of statements relating to good relationships focuses on certain regional peculiarities.

*Availability for Home Visit*

Among respondents who report a good relationship with their GP (mainly in the Calabria region, six cases), older people who could receive medical visits at home mainly emerged. Moreover, when underlining the “goodness” of the current GP who makes home visits, seniors complain about the previous one, mainly because he/she never went to their home, and only wrote prescriptions.

*My GP is really available. When I need something, I call him and he comes home. (CAL_9)*

*Once a month the GP comes to my home, also without announcing this. He measures my blood pressure and visits me. He is a very lovely person! (MAR_39)*

*I have a very good relationship with this GP. He also comes home. The previous one only signed prescriptions. She did not take any responsibility, and eventually suggested to have a specialist visit. (MAR_1)*

*GP Very Careful, not Just Prescriptions*

In two cases in the north, the GP is so caring that (in addition to home visits) he also offers to personally deliver prescriptions to the pharmacist, if necessary. In two very particular, confidential relationships in the south, the GP supports some patients with money.

*My GP is an angel! (…). I call and tell him ‘I need this, this, this and this!’. He makes me the prescription, and if I need, he personally takes it to the pharmacy. (LOM_34)*

*Money I have is not enough! It is mainly used to buy expensive medicines. (…). If I need, I do not ask for anything to my relatives. More than once I have had financial help from my GP! He often supports me! (CAL_17)*

*Nearby Doctor’s Office*

Having the GP’s office nearby also helps the relationship between doctor-patient (two cases in the south), though distance in this regard can also compromise it (as mentioned above).

*My GP lives nearby, few steps away. This is really a great convenience. I have no problems with that. (CAL_7)*

*The office of my GP is close to my home, about 100 m of walking (…). I go to the doctor alone. (CAL_9)*

*When the Good GP Retires*

Sometimes, the relationship with the GPs is so friendly that seniors are concerned about the idea that they may retire, or they regret a recent retirement (although the current doctor is also available), with the consequent loss of a longstanding confidence (two cases).

*I have an excellent GP, reliable and insightful, I am in a crisis at the idea of his retirement! (MAR_18)*

*Since 1994 to last month I had a friendly GP. She assisted my mother and my sister. I also called her in the night and she was always available. Currently she is retired (…). Now I have another GP, who is available to come home when I need. But I miss my previous GP! (CAL_33)*

## 4. Discussion

The aim of this study was to explore how frail older people ageing at home and alone, self-rate their health status; which health services they use; and which barriers hamper their access to them. Overall, our results showed various contexts, with seniors reporting mainly poor health, and barriers for accessing both public and private health services, including the GP. However, some better/fewer situations also emerged, with seniors reporting good health despite having many diseases, and highlighting a good relationship with a caring GP. Results also suggest some regional peculiarities.

### 4.1. Health Status of Older People

In later life, a poor SRH is sometimes correlated with an increased risk of overall decline in physical functions, regardless of the severity of the pathologies [51]. According to EPICENTRO [52], 88% of the Italian population aged over 65 years judged their health overall to be positive (“fair” 49%; “good” or “very good” 40%). The remaining 12% gave instead a negative opinion, reporting their health as “bad” or “very bad”. Older respondents in our study reported mainly poor-unstable health, and also referred to various chronic diseases, e.g., arthritis/osteoporosis; heart disease/hypertension; diabetes and COPD. Results regarding the main chronic diseases are also confirmed by ISTAT [26], with 48% of older people suffering mainly from arthritis and 47% from hypertension. The major question, as highlighted by our interviewees, is when a poor health status compromises mobility and autonomy, especially walking outside home. In this respect, chronic diseases can significantly affect the quality of life of older people, especially when they increase their need for assistance. As evidenced by the Italian Ministry of Health [25], with ageing, chronic diseases become the main cause of morbidity and mortality. Thus, chronicity is associated with the decline of functional capacity and relationships, with a consequent increase in the use of health and social resources. In the IN-AGE sample, the most “critical” subjects are those with greater movement and walking difficulties; some authors also highlighted how decline in functional capabilities and mobility loss (e.g., difficulties in walking or climbing stairs) are associated with negative health outcomes, disability and reduced autonomy [53]. Previous authors [54] stressed in particular how a poor perception of health was associated with reduced walking activity. However, some respondents also perceived good-acceptable health, despite chronic diseases and somatic complaints. These seniors are more optimistic and seem satisfied, given their age, especially if the head “still works”. In particular, older people hope to maintain their cognitive capacity for as long as possible, and in turn the absence of cognitive impairment provides a more positive vision of one’s health conditions. In the literature, cognitive decline was indeed often associated with ageing and reduced quality of life [55,56]; older adults’ perceptions of health were also linked to medical conditions, disability and cognitive performance [57]. It is also worth highlighting the relevance of the adaptive capacity and resilience of frail older people for good ageing. Indeed, personal resources allow seniors to feel they can lead a good quality of life in old age, even though they have negative medical/health outcomes. In this respect, some authors argue that, if on the one hand, older people with worse health conditions run a greater risk for their psychological health [58], on the other hand their long experience of “lived life”, and the need to cope with previous stressful events, can act as a protective/resilience factor [59]. In particular, high resilience has also been significantly associated with longevity and successful ageing [60].

### 4.2. Use of Public and Private Health Services

All the interviewees need the GP, and the MS is fairly frequently requested in both the public and private sector (though slightly more in the latter), followed by a lower use of rehabilitation and nursing services, and diagnostic tests. In this respect, ISTAT [26] confirms the overall use of GP (90%) and MS (66%) by seniors aged 65 years and over. In later life, health needs increase, especially if diseases arise, and the purchase of services on the private market increases as public coverage from the NHS is no longer enough. In particular, according to forecasts up to 2030 [61], total private healthcare spending could reach and exceed 46 billion EUR, largely attributable to older people, giving further confirmation of the link between advancing years, increased healthcare spending, and the increase of its private component. As reported by ISTAT [26], the presence of health problems and the loss of autonomy determine a growth in healthcare consumption, especially after the age of 75, with increasing recourse to MSs and hospital admission. Moreover, frail older people with functional limitations use rehabilitation interventions (as reported by our respondents), which Cowley and colleagues [62] describe as having a great potential for recovery, especially after periods of acute illness or hospitalization. In this regard, a review evaluating 51 articles on the topic [63] highlighted that after hospitalization, 11% of older patients aged 75 years and over, are referred to rehabilitation facilities. 

The need for home health care should also be considered, which in some cases is carried out, for example, for withdrawals; nevertheless, this remains marginal for our respondents. Ranci and colleagues [64] report that in Italy only 5.4% of older people aged over 75 years, and living alone, receive overall home health care. Moreover, some authors [65] have evidenced that home nursing care allows older people to age in place, even though doing so depends on functional limitations, chronic conditions, available care arrangements and income, with the last aspect impacting the possibility of benefiting from private home care. It can also be highlighted that, although it emerged in only one case, older people who live alone, especially when widowed, do not want take care of their health any more as they no longer want to be treated. As Jin and Chrisatakis [66] indicate, the transition to widowhood seems to “distract” people from taking care of themselves (self-neglect), with men in particular suffering a decline in their ability to relate to formal medical contexts.

### 4.3. Barriers Hampering the Use of Health Services

Our results show that, in the public sector, it is mainly long waiting lists that often discourage the use of health services. As a consequence of this, and especially if older people expect too much from a visit or diagnostic test, their health can worsen in the meantime. Seniors could turn to the private health sector, but in this case a major barrier is the cost that emerges exclusively for private services, in particular for MS’s visits. In some cases, it is even necessary to give up healthcare for economic reasons. Indeed, there seems to be an “unspoken” rule, in which, in order to use health services, “you must pay” for a private one, or “you must wait” for a public one. Moreover, for both sectors, the obstacle of the geographical distance between one’s home and the place where the service is provided emerges strongly, in addition to the need for accompaniment and transport, especially in cases of functional limitations of older users. The economic possibility to pay for private transport could thus have a further impact on the use of both private and public health services, as well as the size and proximity of the family network and, therefore, the presence of family members who can “accompany” their elderly relative. 

Literature [67] shows that, in several EU countries, including Italy, Lithuania and Poland, long waiting times and financial barriers, in addition to scarce transport services for reaching distant health facilities, increased disparities in accessing essential healthcare, especially for older people. Also, several ISTAT data [24,30] confirm these findings, with the main overall barriers in 2019 being long waiting times (20%), high costs (10%) and scarce transportation (7%), in addition to further environmental barriers due to difficulty in using transport, and a lack of facilities to access buildings (about 4%). In particular, availability of transport represents a fundamental aspect for health care access, especially for patients with multimorbidity and chronic diseases, since an opposite context may lead to missed appointments and un-appropriate medication use, with consequences such as unmet health needs and poor health outcomes [68].

Regarding environmental barriers, some of our respondents also evidenced that, when they have difficulty in walking, e.g., they use a cane, it is very complicated to take a bus and access buildings with architectural barriers (those without handrails, ramps and elevators). As stressed by Sarlo and colleagues [69], accessible buildings and public transportation can encourage older people to move around. There is indeed a strong link between ageing, disability and architectural/urban design. Also, other authors [70] highlight that architectural barriers represent a strong material and psychological obstacle to older people leaving their homes, and the use of local public transport is perceived by the majority of seniors as dangerous.

Some respondents also reported barriers, such as excessive bureaucracy of various administrative practices and poor treatment affecting in particular the public health sector. Also, Stiglitz and Rosengard [71] highlight how this latter aspect of the public sector is due to critical organizational and bureaucracy procedures. The public sector is not involved in competition for prices and related incentives for improving technical efficiency. Moreover, the greater use of administrative formalities impacts the overall delivery process [72].

### 4.4. The Relationship with GP

In our study, the GP is used by all respondents, mainly for the prescribing of drugs, but also of public MSs visits and diagnostic tests. In Italy, authorization by GPs is necessary in this respect, and they represent the first reference point for health care [73]. GPs are thus the most appropriate healthcare professionals for identifying physical health problems and potential risks, especially for the frail population [16]. There are several reasons that prevent, or in some cases facilitate, the relationship with the GP. A bad relationship is attributed mainly to the “aseptic” and mechanical writing of prescriptions of drugs and medical examinations, and then to a lack of both confidence/trust and home visits. For some, the GP does not seem to play a role in monitoring and supporting frailty in old age. When, conversely, a good relationship with the GP is reported, the possibility of receiving a home visit is the most important reason, in addition to the perception of a very caring GP. 

Benefitting from home visits is thus a crucial issue, especially when seniors have functional limitations and scarce in-person contact with GPs in their medical practice. Often, older patients interact with their family doctor by means of telephone calls, or the relationship is mediated by family members. As stressed by some authors [74], over the last 50 years, GPs visiting patients in their homes has registered a decline internationally; moreover, not all GPs are persuaded that home visits have particular advantages for patients. Conversely, patients consider this practice as improving the relationship. GPs consulting with patients in their homes represents an important element of NHS general practice, especially for frail older people living with multimorbidity and complex needs; moreover, home visits provide “opportunities to develop insights into how illness affects their lives (…). However, with workloads in NHS primary care rising, and increasing pressures on the GP workforce, the place of home visits in core general practice provision is facing increasing challenge” [74] (p. 306). In Italy, in 2018, over a third of GPs exceeded the 1500 assisted threshold; this might indicate a situation of overload of patients, with the consequent implications in terms of loss of efficiency and possible worsening of the quality of care [73]. A study carried out in Germany [75] with 24 GPs, highlighted that most of them were not greatly motivated to undertake home visits, due to workload and low financial reimbursement. Oher authors [76] found that continuity of support and home visits from GP are linked to reduced use of emergency departments, hospitalization and outpatient specialist services. All these aspects should thus be considered and addressed, in order to allow patients to have their desired healthcare, especially in later life. 

Further issues impacting the relationship between GP-patients, i.e., the existence, or not, of confidence/trust between them, and the availability, or not, of a very caring family doctor, are in turn associated with the overall availability to provide home visits, as mentioned above. Croker and colleagues [77] produced evidence that these aspects seem crucial in order to have “effective clinical encounters” [77] (p. 2). They also observed a positive relationship between patients’ trust and their perceptions of communication with their GP, with both a sense of “being taken seriously” and of “shared decision-making” [77] (p. 2) emerging. In turn, a trusting GP-patient relationship encourages the latter to adhere more to medical prescriptions, with consequent improvement of health outcomes. Previous research also reported how a trusting GP-patient relationship is facilitated (among other factors) by adequate consultation length [78], continuity of care [79] and providing overall support [80]. In this respect, our findings highlight in particular that sometimes the relationship with the GPs is so friendly that seniors are concerned about the idea they may retire, with the consequent loss of a longstanding confidence relationship. 

It is also worth mentioning the important role of pharmacists, who are preferred to GPs in some situations (e.g., for blood pressure measurement), whereas others collaborate with the GP in order to provide drugs to older patients. Cittadinanzattiva-Federfarma [81] underlines that the pharmacy is an important health center, especially in the internal/rural areas of Italy, and also for monitoring possible drug interactions. Wood and colleagues [82] reported how seniors positively valued the provision of pharmaceutical care, which contributes to building a trusting relationship, with great importance attributed to “personal and relational factors” and “service awareness” [82] (p. 123).

### 4.5. Some Territorial Differences

With regard to health status, no particular regional differences emerged in our study. Conversely, ISTAT data for Italy in 2019 [26] underlined an overall disadvantage of health indicators in the south, compared to the north. In particular, the share of people aged 75 years and over suffering from three or more chronic diseases is higher in the south (52%, vs. 36% in the north and 43% in the center) [83]. Also, according to EPICENTRO [52], the geographical gradient shows a higher prevalence of older people aged 65 years and over who are satisfied with their health among residents in northern Italy than in the south. In our study, the fact that participants were recruited solely in three Italian regions, in addition to considering north and south only one region for each, could have produced this discrepancy.

With regard to the use of health services, the main results of our study highlight that public visits provided by MSs emerged more in the north, whereas private ones were more in the south. Regarding barriers, they were mainly reported in the south. In particular, for the public sector, long waiting lists and geographical distance were mostly reported, in addition to some cases of poor treatment (e.g., several hours of waiting for treatment in the emergency room, and little attention from medical/nursing staff), and architectural barriers preventing easy entry into health centers. In the private sector, major problems of cost and transport emerged. Similarly, according to ISTAT [24,30] in 2019 in the north and in the south, respectively, about 70% and 60% of seniors used public MSs (totally free or upon payment of a ticket), whereas 33% and 37% used private MSs (totally upon payment). The same ISTAT data indicate that barriers are, overall, more present in the south than in the north (e.g., waiting lists, costs and transportation are reported by 23%, 16% and 11% in the former, and by 16%, 6% and 4% in the latter, respectively). 

This overall picture highlights a better context in the north than in the south, with efficiency and good organization of health services, especially public ones, in the former. Indeed, the Italian NHS presents several differences between Regional Health Systems (RHSs), which lead to welfare territorial inequalities, in particular with regard to accessing health services [84]. In Italy, the NHS allows universal access to healthcare; the Ministry of Health decides on services to be delivered and national funds are allocated to the regions, which administer and plan healthcare activities by organizing supply. However, due to existing differences in this respect, a general strong north-south gradient for healthcare access and utilization emerges, showing a worsening situation in the latter [85]. In Italy, three clusters/levels of RHS performance can be identified, [86]: excellent, including Lombardy, where the health system is reported as one of the best in the country; intermediate, including Marche; and critical, including Calabria. It is also worth underlining that in the north, private healthcare expenditure performs an integrative function of the NHS, whereas in the center-south, it has the main role of reducing waiting lists in the public sector. Overall, due to long public waiting lists, 73% of residents in the south, 69% in the center and 52% in the north turned to the private sector in 2019, CENSIS [61]. Regarding architectural barriers, which were reported only by respondents in the south, it seems that in the other two regions these are not usually perceived as impediments to use the services. According to Martinelli and colleagues [70] this can be attributed to greater attention given to building standards and maintenance in the center-north, than in the south; but it could also depend on the time of construction of the buildings, less recent in the latter. 

Regarding the relationship with the GP, a bad overall context mainly emerged in the Lombardy region. This could be attributed to the fact that this region is among those with the highest percentages of GPs (50%) who crossed the threshold of 1500 patients, with a consequent higher negative impact on efficiency and availability of GPs in this part of the country [73]. Moreover, and probably due also to this “cure burden”, Lombard GPs do not provide home visits very frequently, as reported by our respondents. 

Finally, some seniors interviewed in rural areas reported difficulties in reaching and accessing health services. It is also evidenced that, in some internal municipalities of the south included in the study, the GP sometimes is available only twice a week. Some authors confirm this by reporting that older people living in rural/internal areas (and with low income) often encounter greater barriers in accessing medical care, and this in turn impacts the occurrence of various diseases [87]. ISTAT data [73] indicate that, in the period 2018–2020 (in small municipalities with up to 2000 inhabitants), the percentage of families (not only older people) reporting such difficulties is about 9%, while it halves in urban/metropolitan areas (4%). In this respect, the European Commission [88] highlights that in some EU countries, people mainly living in rural areas called “medical deserts” [88] (p. 18), refer to unmet needs for medical examination, due to problems in accessing healthcare. 

### 4.6. Limitations

The study has some limitations to be considered, in order to have a more accurate interpretation of the findings. Firstly, the sample of respondents were recruited solely in three Italian regions, thus they cannot be considered as representative of the entire Italian situation. This aspect, and the qualitative findings, limit the generalization of the results. The definition of frailty should include several physical, psychological and social domains, but this holistic approach is too complex to be assessed. Thus, for the purpose of the survey, frailty is determined only by an age of 65 years and over, living alone without cohabiting relatives, and the presence of limited functional abilities, thus needing support for the activities of daily living. The cognitive assessment of participants is based only on the information provided by both recruitment channels and respective relatives. The inclusion of seniors with intermediate mobility between limited/reduced within the home, and outside the home (with the support of a person or aids), and thus the exclusion of those with higher physical (and cognitive) limitations (in order to have individuals able to answer questions independently), might have impacted on the low number of older people assisted by PCAs in the final sample. Seniors with overall severe conditions are indeed usually those more often supported by these private assistants. In the Marche region, the choice of inner/rural areas to be included was affected by the earthquake that occurred between August 2016 and January 2017 in central Italy, with hard consequences especially for older people living alone in many rural sites. These zones were thus excluded from the study, in order to have comparable rural areas in the three regions. Furthermore, as the sample is not gender-equitable (90 women and 30 men), the gender insight was not explored, even though this could add possible further reflections. Moreover, the absolute values in tables and figures are to be interpreted with caution, since they are sometimes very low and the sample is not representative. For more details on limitations of the IN-AGE study, further information can be found in a previous publication [42], from which these aspects are partially adapted.

### 4.7. Trustworthiness of the Analysis

Despite some limitations, the trustworthiness of the qualitative analysis in our study is supported by the following four criteria, indicated by Lincoln and Guba [89]: credibility, transferability, dependability and confirmability. The credibility lies on the use of a topic guide that is based on questionnaires largely applied in previous studies on frail, older people needing assistance, e.g., ADLs and IADLs scales [37], and on frequent peer de-briefing discussions among researchers with longstanding expertise on the topic of ageing at home, to define protocol and data collection, rules for conducting analysis, and discussing findings. In addition, by means of dissemination seminars with several stakeholders and experts, the preliminary results were gradually compared and validated. The transferability of qualitative analysis [90] is achieved through a deep literature review as background data [91] and analysis of results from previous studies on the topic e.g., an ISTAT multi-purpose survey [64], which were fundamental for structuring the initial framework [92]. The dependability and confirmability of findings, to be intended as use of replicable methods assuring their duration over time, were gained through an accurate study protocol (approved by a Bioethics Committee), with several notes regarding data collection and analysis, in order to support the adoption of transparent procedures. [91]. Dependability was also based on frequent sessions with researchers and interviewers, in order to have a common vision of data analysis and final dataset [93]. For more details on trustworthiness of the IN-AGE study, further information can be found in a previous publication [42], from which these aspects are partially adapted.

## 5. Conclusions

Overall, our results showed that the majority of frail older people aging in place alone, suffer from physical limitations, perceive a poor health, and report many diseases. Moreover, GPs and MSs are their main “health providers”. Various barriers for accessing health services emerged, especially long waiting lists in the public sector and high costs in the private one, in addition to problems regarding the geographical access to health services for both. In particular, complaints emerged when GPs do not provide home visits. However, a few good situations were referenced, e.g., seniors with good overall health, despite many diseases, and a good/trusting relationship with the GP. Results also suggested some regional peculiarities, with a better overall context in the north than in the south, especially for the public health sector.

These considerations provide useful suggestions/recommendations for health policy makers, mainly regarding the necessity to address the question of long public waiting lists; to provide low-cost private services; to deliver health services close to frail older people; to organize dedicated transport services for them; and to remove architectural barriers in public buildings. In particular, the question of geographical distance can be overcome by creating a widespread network of “community houses”, as planned by the Italian National Recovery and Resilience Plan (NRRP) [94]. These health structures are easily accessible for the community, where GPs, nurses, MSs and other health professionals provide basic health services throughout the territory. Also, it is important to support seniors, and respective family caregivers, with information on the available services, and assistance for the whole access process, for instance, by means of personal navigators (e.g., GP, social worker, community volunteer) [95,96]. Appropriate and barrier-free healthcare services for this population group can thus “make the difference”, in particular, home services can be of great help for ageing at home, without ageing alone. In this respect, it seems necessary to “listen” to the EU carers who consider the most important characteristics of home services to be that “help is provided when it is needed most” (without waiting for too long), and that health professionals are careful and “treat the older person with dignity and respect” [37]. Moreover, a widespread diffusion throughout the national territory of health structures that provide essential services to citizens, is of fundamental importance for the well-being of older residents [88].

Finally, a further issue is the necessity to provide a social-health coordination of the patient’s needs, in order to avoid the risk of both over-treatment (that lengthens the waiting lists for diagnostic procedures and specialist visits for each of the individual pathologies), and under-treatment (the tendency to underestimate certain conditions, e.g., cognitive impairment, which reduces the system’s ability to prevent specific risks). The Italian LTC system is, however, still characterized by high fragmentation in terms of sources of funding, governance and management responsibilities. Indeed, health and social services represent two different and only partially integrated sectors [97]. Conversely, an integrated social-health care provision could allow more appropriate NHS responses and use of resources [25]. One of the most current complex challenges is indeed represented by the optimization and integration of available resources, in order to manage the growth of chronic degenerative diseases, in particular for older groups of the population, assuring them the continuity of assistance for long periods. Finally, a future research direction could take into account the gender insight, in order to explore possible differences, e.g., with regard to both SRH and barriers hampering the use of health services. This could represent an added value and suggest other reflections for/on the topic, thus contributing to gender equality and providing more inclusive and representative research findings.

## Figures and Tables

**Table 1:** Macro-Categories, Sub-Categories and Labels.

Macro-Categories	Sub-Categories	Labels
**Health status** **of older people**	Self-rated health (SRH)	Poor-Unstable; Good-Acceptable
	Main diseases	Arthritis/Osteoporosis; Heart Disease/Hypertension; Diabetes; Asthma/Chronic Obstructive Pulmonary Disease (COPD)
**Health services used** **by older people**	Public and Private Health Services used	General Practitioner (GP, only public); Medical Specialist (MS); Diagnostic Tests; Rehabilitation; Nurse
	Barriers for using services	High cost; Long waiting lists; Architectural barriers; Bureaucracy; Poor treatment; Transport/Distance
**Relationship between** **General Practitioner (GP)** **and older people**	Good relationship	Reasons: available to home visits, GP very careful, nearby doctor’s office
	Bad relationship	Reasons: only prescriptions, lack of confidence/trust, no home visits, no physical visits in office, not present in case of emergency, medical office not/little accessible

**Table 2:** Sample Characteristics (absolute values/*n* and %).

Characteristics	*n* = 120	%
**Age Groups** (years)		
67–74	17	14
75–79	19	16
80–84	28	23
85 and over	56	47
**Gender**		
Male	30	25
Female	90	75
**Education**		
No title	14	12
Primary school (5 years)	55	46
Middle school (3 years)	20	17
High school (3–5 years)	28	23
University/similar (3–5 years)	3	2
**Marital Status**		
Single	16	13
Married but not cohabiting	2	2
Divorced/separated	14	12
Widowed	88	73
**Living Situation**		
Alone	93	78
With personal care assistant (PCA)	27	22
**Level of physical limitations ^1^**		
Mild	30	25
Moderate	33	28
High	27	23
Very high	30	24
**Total Cases/Respondents**	120	100

^1^ The level of physical/functional limitations is based on 12 ADLs-IADLs, two mobility limitations (going up/down the stairs and bending to pick up an object), plus sensory limitations in hearing and seeing. Mild = no activities “not able”; Moderate = one-two; High = three-four; Very high = five or more.

## Data Availability

The quantitative data supporting the findings of the study are not publicly available due to privacy/ethical restrictions. There is indeed confidential information that could compromise the anonymity of research participants as potential indirect identifiers, e.g., city of residence of respondents. Also, original verbatim transcriptions in the charts are not publicly available due to privacy/ethical restrictions, that is to their containing information that could compromise the privacy/anonymity of research participants. (e.g., including names and locations and other potential identifiers of respondents). However, a dataset regarding the sample of the main study carried out in 2019, is openly available in Mendeley at https://doi.org/10.17632/3ryrpz224h.2 (accessed on 20 June 2022).

## References

[1] ISTAT (2022). Popolazione Italiana Residente al 1° Gennaio, 2022.

[2] Ricciardi W., Tarricone R. (2021). The evolution of the Italian National Health Service. Lancet.

[3] EUROSTAT (2022). Population Structure and Ageing.

[4] ISTAT (2021). Aspetti Della Vita Quotidiana. Famiglie, Persone Sole.

[5] Melchiorre M.G., Quattrini S., Lamura G., Socci M. (2022). Role and Characteristics of Personal Care Assistants of Frail Older People with Functional Limitations Ageing in Place in Italy. Int. J. Environ. Res. Public Health.

[6] Mitnitski A.B., Graham J.E., Mogilner A.J., Rockwood K. (2002). Frailty, fitness and late-life mortality in relation to chronological and biological age. BMC Geriatr..

[7] Wleklik M., Uchmanowicz I., Jankowska E.A., Vitale C., Lisiak M., Drozd M., Pobrotyn P., Tkaczyszyn M., Lee C. (2020). Multidimensional Approach to Frailty. Front. Psychol..

[8] Pilotto A., Custodero C., Maggi S., Polidori M.C., Veronese N., Ferrucci L. (2020). A multidimensional approach to frailty in older people. Ageing Res. Rev..

[9] Rajabali N., Rolfson D., Bagshaw S.M. (2016). Assessment and utility of frailty measures in critical illness, cardiology, and cardiac surgery. Can. J. Cardiol..

[10] Vermeulen J., Neyens J.C., Van Rossum E., Spreeuwenberg M.D., De Witte L.P. (2011). Predicting ADL disability in community dwelling elderly people using physical frailty indicators: A systematic review. BMC Geriatr..

[11] Jędrzejczyk M., Foryś W., Czapla M., Uchmanowicz I. (2022). Relationship between Multimorbidity and Disability in Elderly Patients with Coexisting Frailty Syndrome. Int. J. Environ. Res. Public Health.

[12] Ofori-Asenso R., Chin K.L., Curtis A.J., Zomer E., Zoungas S., Liew D. (2019). Recent Patterns of Multimorbidity among Older Adults in High-Income Countries. Popul. Health Manag..

[13] Yarnall A.J., Sayer A.A., Clegg A. (2017). New horizons in multimorbidity in older adults. Age Ageing.

[14] Molist-Brunet N., Sevilla-Sánchez D., Puigoriol-Juvanteny E., Bajo-Peñas L., Cantizano-Baldo I., Cabanas-Collell L., Espaulella-Panicot J. (2022). Individualized Medication Review in Older People with Multimorbidity: A Comparative Analysis between Patients Living at Home and in a Nursing Home. Int. J. Environ. Res. Public Health.

[15] Stafford G., Villén N., Roso-Llorach A., Troncoso-Mariño A., Monteagudo M., Violán C. (2021). Combined Multimorbidity and Polypharmacy Patterns in the Elderly: A Cross-Sectional Study in Primary Health Care. Int. J. Environ. Res. Public Health.

[16] Rodríguez-Laso A., Caballero Mora M.A., García Sánchez I., Alonso Bouzón C., Rodríguez Mañas L., Bernabei R., Gabrovec B., Hendry A., Liew A., O’Caoimh R. (2019). Updated State of the Art Report on the Prevention and Management of Frailty.

[17] Herndon L.A., Schmeissner P.J., Dudaronek J.M., Brown P.A., Listner K.M., Sakano Y., Paupard M.C., Hall D.H., Driscoll M. (2002). Stochastic and genetic factors influence tissue-specific decline in ageing C. Elegans. Nature.

[18] Pasanen T.P., Tamminen N., Martelin T., Mankinen K., Solin P. (2021). Profiles of subjective health among people living alone: A latent class analysis. BMC Public Health.

[19] Li C.M., Lin C.H., Li C.I., Liu C.S., Lin W.Y., Li T.C., Lin C.C. (2021). Frailty status changes are associated with healthcare utilization and subsequent mortality in the elderly population. BMC Public Health.

[20] Beard J.R., Bloom D.E. (2015). Towards a comprehensive public health response to population ageing. Lancet.

[21] Chimento-Díaz S., Sánchez-García P., Franco-Antonio C., Santano-Mogena E., Espino-Tato I., Cordovilla-Guardia S. (2022). Factors Associated with the Acceptance of New Technologies for Ageing in Place by People over 64 Years of Age. Int. J. Environ. Res. Public Health.

[22] Hoeck S., Francois G., Geerts J., Van der Heyden J., Vandewoude M., Van Hal G. (2012). Health-care and home-care utilization among frail elderly persons in Belgium. Eur. J. Public Health.

[23] Hajek A., Bock J.O., Saum K.U., Matschinger H., Brenner H., Holleczek B., Haefeli W.E., Heider D., König H.H. (2018). Frailty and healthcare costs-longitudinal results of a prospective cohort study. Age Ageing.

[24] Peters L.L., Burgerhof J.G., Boter H., Wild B., Buskens E., Slaets J.P. (2015). Predictive validity of a frailty measure (GFI) and a case complexity measure (IM-E-SA) on healthcare costs in an elderly population. J. Psychosom. Res..

[25] Italian Ministry of Health (2020). Invecchiamento Della Popolazione e Sostenibilità del SSN.

[26] ISTAT (2021). Le Condizioni di Salute Della Popolazione Anziana in Italia, Anno 2019.

[27] ISTAT (2019). Annuario Statistico Italiano.

[28] ISTAT (2017). Anziani: Le Condizioni di Salute in Italia e Nell’unione Europea. Anno 2015.

[29] ISTAT (2017). Condizioni di Salute e Ricorso ai Servizi Sanitari in Italia e Nell’unione Europea. Indagine EHIS 2015.

[30] ISTAT (2021). Condizioni di Salute e Ricorso ai Servizi Sanitari in Italia e Nell’unione Europea. Indagine EHIS 2019.

[31] De Rossi A. (2020). Riabitare l’Italia. Aree Interne Tra Abbandoni e Riconquiste.

[32] OECD (2020). OECD Regions and Cities at a Glance 2020.

[33] NSIA—National Strategy for Inner Areas (2018). Annual Report on the National Strategy for Inner Areas.

[34] ISTAT (2020). Le Misure Della Vulnerabilità: Un’applicazione a Diversi Ambiti Territoriali.

[35] Ritchie J., Lewis J. (2003). Qualitative Research Practice. A Guide for Social Science Students and Researchers.

[36] Colombo F., Llena-Nozal A., Mercier J., Tjadens F. (2011). Help Wanted? Providing and Paying for Long-Term Care.

[37] Lamura G., Dohner H., Kofhal C. (2008). Supporting Family Carers of Older People in Europe—Empirical Evidence, Policy Trends and Future Perspectives.

[38] Katz S. (1983). Assessing Self-Maintenance: Activities of Daily Living, Mobility, and Instrumental Activities of Daily Living. J. Am. Geriatr. Soc..

[39] ISTAT (2019). Conoscere il Mondo Della Disabilità: Persone, Relazioni e Istituzioni.

[40] ISTAT (2011). Indagine Statistica Multiscopo Sulle Famiglie.

[41] European Union (2016). Regulation 2016/679 of the European parliament and of the Council. General data protection regulation. Off. J. Eur. Union..

[42] Melchiorre M.G., Quattrini S., Lamura G., Socci M. (2021). A Mixed-Methods Analysis of Care Arrangements of Older People with Limited Physical Abilities Living Alone in Italy. Int. J. Environ. Res. Public Health.

[43] Srivastava A., Thomson S.B. (2009). Framework Analysis: A Qualitative Methodology for Applied Policy Research. J. Adm. Gov..

[44] Ritchie J., Spencer L., Bryman A., Burgess R.G. (1994). Qualitative data analysis for applied policy research. Analyzing Qualitative Data.

[45] Mayring P. (2000). Qualitative Content Analysis. Forum Qual. Soc. Res..

[46] Saldana J. (2009). The Coding Manual for Qualitative Researchers.

[47] Weitzman E.A., Denzin N.K., Lincoln Y.S. (2000). Software and qualitative research. Handbook of Qualitative Research.

[48] Gale N.K., Heath G., Cameron E., Rashid S., Redwood S. (2013). Using the Framework Method for the Analysis of Qualitative Data in Multi-Disciplinary Health Research. BMC Med. Res. Methodol..

[49] Bree R., Gallagher G. (2016). Using Microsoft Excel to Code and Thematically Analyse Qualitative Data: A Simple, Cost-Effective Approach. AISHE J..

[50] Corden A., Sainsbury R. (2006). Using Verbatim Quotations in Reporting Qualitative Social Research: Researchers’ Views.

[51] Gyasi R.M., Phillips D.R. (2018). Gender, self-rated health and functional decline among community-dwelling older adults. Arch. Gerontol. Geriatr..

[52] EPICENTRO—Epidemiology for Public Health (2020). La Sorveglianza Passi d’Argento. I Dati per l’Italia. Percezione Dello Stato di Salute.

[53] Billot M., Calvani R., Urtamo A., Sánchez-Sánchez J.L., Ciccolari-Micaldi C., Chang M., Roller-Wirnsberger R., Wirnsberger G., Sinclair A., Vaquero-Pinto N. (2020). Preserving Mobility in Older Adults with Physical Frailty and Sarcopenia: Opportunities, Challenges, and Recommendations for Physical Activity Interventions. Clin. Interv. Aging.

[54] Talkowski J.B., Brach J.S., Studenski S., Newman A.B. (2008). Impact of health perception, balance perception, fall history, balance performance, and gait speed on walking activity in older adults. Phys. Ther..

[55] Muir S.W., Gopaul K., Odasso M.M.M. (2012). The role of cognitive impairment in fall risk among older adults: A systematic review and meta-analysis. Age Ageing.

[56] Qiu Y., Wei K., Zhu L., Wu D., Jiao C. (2021). The Association of Meteorological Factors with Cognitive Function in Older Adults. Int. J. Environ. Res. Public Health.

[57] Warmoth K., Tarrant M., Abraham C., Lang I.A. (2016). Older adults’ perceptions of ageing and their health and functioning: A systematic review of observational studies. Psychol. Health Med..

[58] Kwan C., Walsh C.A. (2017). Seniors’ disaster resilience: A scoping review of the literature, in International. Int. J. Disaster Risk Reduct..

[59] Knight B.G., Gatz M., Heller K., Bengtson V.L. (2000). Age and emotional response to the Northridge earthquake: A longitudinal analysis. Psychol. Aging.

[60] MacLeod S., Musich S., Hawkins K., Alsgaard K., Wicker E.R. (2016). The impact of resilience among older adults. Geriatr. Nurs..

[61] CENSIS (2017). VII Rapporto Sulla Sanità Pubblica, Privata, e Intermediata.

[62] Cowley A., Goldberg S.E., Gordon A.L., Logan P.A. (2021). Rehabilitation potential in older people living with frailty: A systematic mapping review. BMC Geriatr..

[63] Tijsen L.M., Derksen E.W., Achterberg W.P., Buijck B.I. (2019). Challenging rehabilitation environment for older patients. Clin. Interv. Aging.

[64] Ranci C., Arlotti M., Bernardi L., Melchiorre M.G. (2020). La solitudine dei numeri ultimi. Abit. Anziani.

[65] Cho S.H. (2005). Older people’s willingness to use home care nursing services. J. Adv. Nurs..

[66] Jin L., Chrisatakis N.A. (2009). Investigating the Mechanism of Marital Mortality Reduction: The Transition to Widowhood and Quality of Health Care. Demography.

[67] Sowa-Kofta A., Marcinkowska I., Ruzik-Sierdzińska A., Mackevičiūtė R. (2021). Ageing Policies. Access to Services in Different Member States.

[68] Syed S.T., Gerber B.S., Sharp L.K. (2013). Traveling towards disease: Transportation barriers to health care access. J. Commun. Health.

[69] Sarlo A., Bagnato F., Martinelli F. (2019). Ageing in place and the built environment. Implications for the quality of life and the risks of isolation of frail older people. DAStU Work. Pap. Ser..

[70] Martinelli F., Cilio A., Vecchio Ruggeri S. (2021). Ageing in place e contesto abitativo. I condizionamenti dell’ambiente costruito sulla qualità della vita e sui rischi di isolamento degli anziani fragili che invecchiano soli a casa propria: Barriere, mobilità, socialità. DAStU Work. Pap. Ser..

[71] Stiglitz G.E., Rosengard J.K. (2015). Economics of the Public Sector. Fourth Edition.

[72] Banzon E., Mailfert M. (2018). Overcoming Public Sector Inefficiencies toward Universal Health Coverage. ADB Work. Pap. Ser..

[73] ISTAT (2021). Rapporto BES 2020: Il Benessere Equo e Sostenibile in Italia.

[74] Mitchell S., Hillman S., Rapley D., Gray S., Dale J. (2020). GP home visits: Essential patient care or disposable relic?. Br. J. Gen. Pract..

[75] Theile G., Kruschinski C., Buck M., Müller C.A., Hummers-Pradier E. (2011). Home visits—Central to primary care, tradition or an obligation? A qualitative study. BMC Fam. Pract..

[76] Hansen A.H., Kristoffersen A.E., Lian O.S., Halvorsen P.A. (2014). Continuity of GP care is associated with lower use of complementary and alternative medical providers: A population-based cross-sectional survey. BMC Health Serv. Res..

[77] Croker J.E., Swancutt D.R., Roberts M.J., Abel G.A., Roland M., Campbell J.L. (2013). Factors affecting patients’ trust and confidence in GPs: Evidence from the English national GP patient survey. BMJ Open.

[78] Freeman G.K., Horder J.P., Howie J.G., Hungin A., Hill A.P., Shah N.C., Wilson A. (2002). Evolving general practice consultation in Britain: Issues of length and context. BMJ.

[79] Tarrant C. (2010). Continuity and trust in primary care: A qualitative study informed by game theory. Ann. Fam. Med..

[80] Cocksedge S., Greenfield R., Nugent G.K., Chew-Graham C. (2011). Holding relationships in primary care: A qualitative exploration of doctors’ and patients’ perceptions. Br. J. Gen. Pract..

[81] Cittadinanzattiva-Federfarma (2019). Rapporto Annuale Sulla Farmacia. II^a^ Edizione.

[82] Wood K., Gibson F., Radley A., Williams B. (2015). Pharmaceutical care of older people: What do older people want from community pharmacy?. Int. J. Pharm. Pract..

[83] ISTAT (2020). Aspetti di Vita Degli over 75.

[84] Giarelli G., Vicarelli G., Vicarelli G., Giarelli G. (2021). Conclusioni. Una bussola per il rilancio del SSN. Il Servizio Sanitario Nazionale e la Pandemia da Covid-19. Problemi e Proposte.

[85] Matranga D., Maniscalco L. (2022). Inequality in Healthcare Utilization in Italy: How Important Are Barriers to Access?. Int. J. Environ. Res. Public Health.

[86] Spandonaro F., D’Angela D. (2017). Una Misura di Performance dei SSR, V Edizione.

[87] Fisher O., Fabbietti P., Lamura G. (2021). Socio-Economic Predictors of Hiring Live-In Migrant Care Workers to Support Community Dwelling Older Adults with Long-Term Care Needs: Recent Evidence from a Central Italian Region. Sustainability.

[88] European Commission (2021). Green Paper on Ageing.

[89] Lincoln Y.S., Guba E.G. (1985). Naturalistic Inquiry.

[90] Polit D.F., Beck C.T. (2010). Generalization in Quantitative and Qualitative Research: Myths and Strategies. Int. J. Nurs. Stud..

[91] Cho J.Y., Lee E. (2014). Reducing Confusion about Grounded Theory and Qualitative Content Analysis: Similarities and Differences. Qual. Rep..

[92] Costa G., Melchiorre M.G., Arlotti M. (2020). Ageing in place in different care regimes. The role of care arrangements and the implications for the quality of life and social isolation of frail older people. DAStU Work. Pap. Ser..

[93] Hagen B.N.M., Sawatzky A., Harper S.L., O’Sullivan T.L., Jones-Bitton A. (2021). What Impacts Perceived Stress among Canadian Farmers? A Mixed-Methods Analysis. Int. J. Environ. Res. Public Health.

[94] Pesaresi F. Le Case della Comunità: Cosa Prevede il PNRR. Welforum.it 2021. https://welforum.it/il-punto/verso-un-welfare-piu-forte-ma-davvero-coeso-e-comunitario/le-case-della-comunita-cosa-prevede-il-pnrr/.

[95] Gwyther H., Shaw R.L., Jaime Dauden E., D’avanzo B., Kurpas D., Bujnowska-Fedak M.M., Kujawa T., Marcucci M., Cano A., Holland C. (2018). Understanding frailty: A qualitative study of European healthcare policy-makers’ approaches to frailty screening and management. BMJ Open.

[96] European Commission and Social Protection Committee (2021). Long-Term Care Report. Trends, Challenges and Opportunities in an Ageing Society.

[97] Melchiorre M.G., Quattrini S., Papa R., Barbabella F., Zanetti E., Lamura G. (2014). Caring for People with Multiple Chronic Conditions in Italy: Policy and Practices.

